# Intracellular delivery of HA1 subunit antigen through attenuated *Salmonella* Gallinarum act as a bivalent vaccine against fowl typhoid and low pathogenic H5N3 virus

**DOI:** 10.1186/s13567-017-0446-1

**Published:** 2017-08-07

**Authors:** Nitin Machindra Kamble, Kim Je Hyoung, John Hwa Lee

**Affiliations:** College of Veterinary Medicine, Chonbuk National University, Iksan Campus, Jeonju, 570-752 Republic of Korea

## Abstract

Introduction of novel inactivated oil-emulsion vaccines against different strains of prevailing and emerging low pathogenic avian influenza (LPAI) viruses is not an economically viable option for poultry. Engineering attenuated *Salmonella* Gallinarum (*S.* Gallinarum) vaccine delivering H5 LPAI antigens can be employed as a bivalent vaccine against fowl typhoid and LPAI viruses, while still offering economic viability and sero-surveillance capacity. In this study, we developed a JOL1814 bivalent vaccine candidate against LPAI virus infection and fowl typhoid by engineering the attenuated *S.* Gallinarum to deliver the globular head (HA1) domain of hemagglutinin protein from H5 LPAI virus through pMMP65 constitutive expression plasmid. The important feature of the developed JOL1814 was the delivery of the HA1 antigen to cytosol of peritoneal macrophages. Immunization of chickens with JOL1814 produced significant level of humoral, mucosal, cellular and IL-2, IL-4, IL-17 and IFN-γ cytokine immune response against H5 HA1 and *S.* Gallinarum antigens in the immunized chickens. Post-challenge, only the JOL1814 immunized chicken showed significantly faster clearance of H5N3 virus in oropharyngeal and cloacal swabs, and 90% survival rate against lethal challenge with a wild type *S*. Gallinarum. Furthermore, the JOL1814 immunized were differentiated from the H5N3 LPAI virus infected chickens by matrix (M2) gene-specific real-time PCR. In conclusion, the data from the present showed that the JOL1814 can be an effective bivalent vaccine candidate against H5N3 LPAI and fowl typhoid infection in poultry while still offering sero-surveillance property against H5 avian influenza virus.

## Introduction

*Salmonella enterica serovar* Gallinarum (*S.* Gallinarum) and avian influenza virus (AIV) are two contagious and infectious pathogens that are responsible for severe economic distress in poultry production [1, 2]. *S.* Gallinarum, an etiological agent of fowl typhoid (FT), causes a severe systemic disease with a high mortality rate in chickens. Similarly, infection of chickens with AIV causes either a mortality or respiratory distress with serious complications depending on the pathogenicity of the infecting virus [1, 2]. AIV is categorized as high-pathogenicity avian influenza (HPAI) or low-pathogenicity avian influenza (LPAI) based on the pathogenicity and virulence in chickens [3]. The HPAI and LPAI viruses cause acute systemic disease with high flock mortality and mild respiratory disease, respectively [3]. The LPAI viruses are widespread around the globe since mid-nineties. According to OIE, from 2006 to 2014, the LPAI H9N2 virus incidences in domestic poultry were notified regularly from the Republic of Korea [4]. Since 2007, the Korean veterinary authority has permitted the use of inactivated H9N2 LPAI vaccine to control the disease [5]. Apart from LPAI H9N2 infection, during 2007–2010, the Republic of Korea has notified four H5 and twenty H7 LPAI subtype virus outbreaks with subclinical infection to the OIE. These regular outbreaks of LPAI viruses in poultry with detection of the H5 and H7 subtypes have raised the concerns about the possibility of emergence of HPAI viruses from pre-circulating LPAI virus in the poultry [6, 7]. Therefore, the implementation of the vaccination strategy for control and prevention of LPAI H5 and H7 subtype viruses infection in poultry are warranted.

Routine vaccination of chickens against *S.* Gallinarum and influenza viruses are the principle means to control the infection and subsequent outbreak of FT and AIV infection [4, 8]. We previously developed an attenuated *S.* Gallinarum vaccine candidate, JOL967 (Δ*lon* Δ*cpxR*), that effectively protected chickens against FT infection [9]. However, stamping out is the preferred method to control HPAI infection, whereas vaccination is principally directed against LPAI virus control. Vaccination programs to control LPAI virus outbreaks are specifically directed against a particular subtype prevalent in a particular area. Moreover, the possibility of HPAI emerging from LPAI increases the importance of vaccinating against LPAI [3, 7, 10]. The prophylactic use of traditional inactivated oil-emulsion vaccines to prevent infection with different LPAI subtypes is efficient but should possess capacity to differentiate between vaccinated and infected birds (DIVA) [11]. Moreover, developing and introducing a vaccine candidate against each LPAI virus subtype is not an economically viable option for poultry production. Therefore, a cost-effective vaccine candidate to control different subtypes of the LPAI virus without interfering with HPAI virus sero-surveillance is needed.

Among the new vaccine platforms, live bacterial vaccine vectors (LBVs) are a promising approach to express and deliver immunogenic heterologous proteins of AIV [12]. LBV-based vaccine development is highly cost effective and allows for a quick development of strain-matched vaccine against the novel circulating influenza viruses, as it circumvents the need for a constant supply of eggs [12]. Recently, an attenuated *Salmonella*-based LBV vaccine system has generated a new perspective for delivering heterologous antigenic proteins [13]. Further, using routine vaccination with *S.* Gallinarum to deliver an LPAI vaccine can reduce the cost and facilitate mass-scale vaccine production [12, 14]. In addition, engineering an *S.* Gallinarum-based LBV system to control LPAI virus infection in poultry has an added advantage in simultaneously protecting chickens against FT. Another characteristic of bacterial vaccine vectors is ease of administration, along with humoral immunity generation of the mucosal and innate immune response against invading pathogens [15].

Hence, we hypothesized that immunization with a bivalent vaccine is a novel approach to simultaneously control bacterial origin FT and H5N3 LPAI virus infection in poultry. A bivalent vaccine candidate was constructed by engineering the *S.* Gallinarum vaccine to carry the globular head (HA1) domain of hemagglutinin from H5 LPAI virus. The candidate was evaluated for its potential to induce immunogenicity and protect against fowl typhoid and LPAI H5 virus infection in a chicken model. Further, as only HA1 gene were used to construct the LBV, the DIVA capability of the LBV was validated by matrix (M2) gene specific real-time PCR.

## Materials and methods

### Construction of *S*. Gallinarum strain JOL967 expressing HA1 from the H5N3 virus

**Figure 1:**
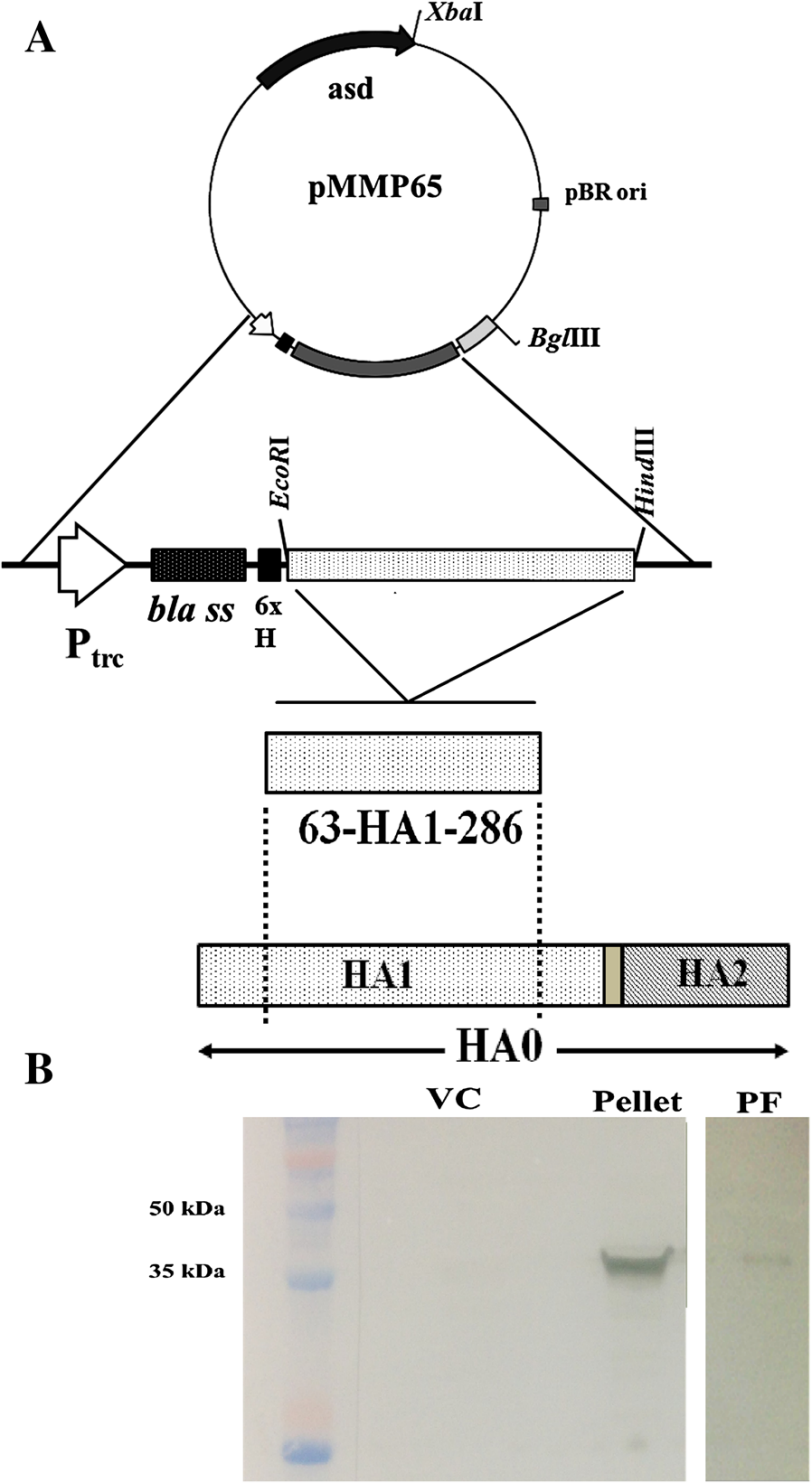
**Schematics for construction of the pMMP65-HA1 constitutive expression plasmid and Western blot analysis of JOL1814. A** Cloning of the HA1 gene (amino acid residues 63–286) from influenza. A H5N3 virus into pMMP65 under a constitutive P_trc_ promoter in frame with the *bla* periplasmic signal sequence. **B** Detection of HA1 protein expressed and secreted by JOL1814 with H5N1, a hemagglutinin (HA) (bird flu) polyclonal antibody. Lane M, size marker; VC: JOL1820 vector control; Pellet: a cellular pellet of JOL1814; PF: periplasmic fraction of JOL1814.

The bacterial and viral strains used in this study are listed in Table 1. An attenuated auxotrophic mutant strain of *S*. Gallinarum, JOL967, was used as a delivery vehicle for the HA1 protein of the influenza A virus H5 subtype. JOL967 was constructed by deleting the *lon*, *cpxR*, and *asd* genes from wild-type *S.* Gallinarum JOL394, as previously described [16]. A computational, *codon*-*optimized*, *synthetic* HA1 gene fragment from the H5N1 and H5N8 subtype of influenza A virus was cloned in pMMP65, an Asd^+^ constitutive expression vector (Figure 1A). The pMMMP65-HA1 plasmid was introduced into JOL967 by electroporation, designated JOL1814, and used for the chicken immunization study. The pMMP65 plasmid was electroporated into JOL967. A positive clone was coined JOL1820 and used as a vector control. The HA1 protein used for ELISA were prepared by cloning codon-optimized, synthetic HA1 gene fragment from the H5N1 and H5N8 subtype of influenza A virus in pET28a (+) plasmid and expressed in BL21 cells as 6× histidine (His) tagged HA1 protein. The BL21 expressed 6× His tagged HA1 protein were purified by affinity chromatography with Ni–NTA agarose column as per manufacturer’s instruction (Quigen, USA).

**Table 1:** **List of plasmid, bacterial and viral strains used**

Strains/plasmid	Description	Reference
*S.* Gallinarum		
JOL967	*∆lon*, *∆cpxR*, *∆asd* mutant of *S.* Gallinarum	[16]
JOL394	Wild-type *S*. Gallinarum from chicken with fowl typhoid	
JOL-1814	JOL967 with pMMP65-HA1 plasmid	This study
JOL1820	JOL967 with pMMP65 plasmid	This study
BL21	*E. coli* cells for protein expression	
H5N3 virus	Influenza A/spot-billed duck/Korea/KNU SYG06/2006 LPAI	
Plasmids		
pMMP65	Asd+, pBR *ori*, -lactamase signal sequence-based periplasmic secretion plasmid, 6 His tag	
pMMP65-HA1	pMMP65 harboring HA1 gene of H5N3 virus	This study
pET28a(+) HA1	pET28(+) harborunf HA1 gene of H5N3 virus	

The strains were maintained as frozen glycerol cultures in LB broth at −70 °C.

### Western blot analysis

Periplasmic expression of the H5 HA1 gene in JOL1814 was confirmed by Western blot assay. To express H5 HA1 protein, JOL1814 was cultured in Luria–Bertani (LB) broth (Becton, Dickinson and Company, USA) until the OD_600_ reached 0.8, after which the pellet was harvested by centrifugation at 3400*g* for 20 min. The periplasmic protein fraction was prepared from harvested cell pellets by the lysozyme-osmotic shock method, as described elsewhere [17, 18]. Boiled protein samples from the pellet and periplasmic fraction were separated by 12% sodium dodecyl sulfate–polyacrylamide gel electrophoresis and transferred to polyvinylidene fluoride membranes (Millipore, USA). The membranes were blocked with 3% bovine serum albumin. Mouse influenza A avian H5N1 hemagglutinin (HA) (bird flu) polyclonal antibody (Cat. No. MBS396043; MyBioSource, USA) and horseradish peroxidase (HRP)-conjugated goat anti-mouse IgY antibodies (Cat. No. NB730-H; Novus Biologicals, USA) were used as primary and secondary antibodies, respectively. The Western blots were developed by adding 3,3′-diaminobenzidine and 4-chloro-1-naphthol (Sigma) in the presence of H_2_O_2_.

### Internalization of JOL1814 into avian peritoneal macrophages

Avian peritoneal exudate or macrophage cells were isolated for gentamicin protection assay, as described elsewhere [19, 20]. The 6-weeks-old chicken was injected intraperitoneally with Sephadex particles. Four days later, the peritoneal macrophages were collected, washed and incubated in six-well plates at 40 °C for 30 min. The non-adherent cells were washed away with Hanks solution and adherent cell monolayer were harvested for internalization assay. The live dead percentage in the scraped adherent peritoneal macrophages were estimated by trypan blue dye exclusion technique. The adherent peritoneal macrophages were seeded on gelatin-coated coverslips at a density of 5 × 10^5^ cells per well in a six-well plate and were infected with JOL1814 and JOL1820 at a multiplicity of infection (MOI) of 100 for 20 min. Then, the infected peritoneal macrophages were washed three times with PBS and incubated with RPMI-1640 supplemented with 50 µg/mL gentamicin for 24 h. After incubation, the cells were fixed with 4% paraformaldehyde and permeabilized with 0.2% Triton-x. The infected cells were incubated with chicken anti-*S.* Gallinarum polyclonal antibody and mice anti-hemagglutinin (HA) H5N1 (Bird Flu) Polyclonal Antibody (Cat. No. MBS396043; MyBioSource, USA). Post-washing, the infected peritoneal macrophages were stained with anti-chicken-fluorescein isothiocyanate and anti-mice alexa-fluor monoclonal secondary antibodies. Finally, the cells were incubated for 10 min with 4 s, 6-diamidino-2-phenylindole nuclear stain and observed under a fluorescence microscope.

### Chicken experiments

The chicken experiments were approved by the Chonbuk National University Animal Ethics Committee (CBU 2014-1-0038) and were performed according to the guidelines of the Korean Council on Animal Care. Female, 1-day-old layer chickens (Brown Nick) were procured and provided antibiotic-free food and water ad libitum. Prior to immunizing the chickens with JOL1814, they were evaluated for the presence of pre-existing *S.* Gallinarum-specific antibodies by ELISA. JOL1814 was cultured in LB broth at 37 °C to an OD_600_ of 0.6 and adjusted to an appropriate concentration in sterile PBS (pH 7.4) before being administered to chickens. The chickens were immunized with JOL1814 and JOL1820 with a prime-boost immunization strategy. The chickens were primed at 4 weeks of age and boosted at 8 weeks of age. The chickens (*n* = 60) were divided into four equal groups (*n* = 20). One group was immunized orally (oral) with 10^8^ colony forming units (CFU) of JOL1814, and another group was immunized intramuscularly (IM) with 10^6^ CFU of JOL1814. The other two groups were inoculated intramuscularly with JOL1820 or sterile phosphate-buffered saline (PBS) and designated as vector and PBS controls, respectively.

### Indirect enzyme-linked immunosorbent assay (ELISA)

Plasma and intestinal lavage samples were collected as described elsewhere [21]. An indirect ELISA was performed to determine the concentrations of plasma IgY and secretory IgA (sIgA) specific for *S.* Gallinarum and H5 HA1 protein. An antigenic outer membrane protein (OMP) extracted from JOL394 *S.* Gallinarum and BL21 expressed 6× His tagged H5 HA1 protein were used separately to coat 96-well MICROLON^®^ ELISA plates (Greiner Bio-One GmbH, Germany). Plasma and intestinal lavage samples were diluted to 1:100 and 1:3 to examine the IgY and sIgA titers, respectively. Goat anti-chicken IgY HRP-conjugate (Southern Biotech, USA) and goat anti-chicken IgA HRP-conjugate (ThermoFisher Scientific, USA) at 1:100 000 dilutions were used as secondary antibodies. The HRP activity of bound secondary antibody was determined with *o*-phenylenediamine dihydrochloride substrate (Sigma-Aldrich, USA). All assays were performed in triplicate. The plasma IgY and intestinal sIgA responses against *S.* Gallinarum were quantified by plotting a standard curve of ODs against a reference antibody concentration using a chicken IgY and IgA ELISA quantitation kit (Bethyl Laboratories, Montgomery, TX, USA), as per the manufacturer’s instruction and described elsewhere [22].

### Lymphocyte proliferation assay (LPA)

Peripheral blood mononuclear cells (PBMCs) were isolated as described previously [23]. Harvested PBMCs were incubated in 96-well tissue culture plates at a concentration of 1 × 10^6^ cells/mL in the RPMI-1640 medium. PBMCs were stimulated with a sonicated bacterial cell protein (sbcp) suspension prepared from JOL394 and purified H5 HA1 protein antigen in a separate PBMC culture for 72 h at 40 °C [24]. Proliferation of stimulated PBMCs was measured with thiazolyl blue tetrazolium bromide dye (Sigma-Aldrich, USA) according to the manufacturer’s protocol. The blastogenic response was expressed as the mean stimulation index (SI), as described elsewhere [25].

### Quantitative analysis of cytokine mRNA level by real-time PCR (qRT-PCR)

PBMCs were harvested from blood samples collected from the JOL1814, JOL1820-inoculated, and PBS control groups at 7 weeks of age following primary inoculation. A total of 1 × 10^6^ viable PBMCs were cultured in six-well tissue culture plates. The cultured PBMCs were stimulated with 4 µg/mL of H5 HA1 protein. The stimulated PBMCs cultures were incubated at 40 °C for 72 h. Total RNA isolated from the stimulated PBMCs were reverse transcribed into cDNA with a QuantiTect Reverse Transcription Kit (Qiagen, USA). The change in cytokine mRNA level was calculated by the 2^−∆∆Ct^ method [26]. The change in mRNA level in immunized chickens was calculated as a fold difference over the PBS control group after normalizing ct value against endogenous control β-actin.

### Hemagglutinin inhibition assay

The haemagglutination inhibition (HI) assay was performed to evaluate functional antibodies in the sera of immunized and control chickens. Heat-inactivated (56 °C for 30 min) serum samples were serially diluted and incubated with 4 HA units of H5N3 virus for 30 min in a V-bottom microtiter plate. Further, chicken red blood cells (RBCs) were added to each well and incubated for 30 min at room temperature. The HAI titer was calculated as the reciprocal of the highest serum dilution that completely inhibited haemagglutination in chicken RBCs. Individual HAI antibody titers were transformed to log_2_ values, and average GMTs were calculated.

### DIVA and determination of protective efficacy

An influenza A/spot-billed duck/Korea/KNU SYG06/2006(H5N3) LPAI virus isolated previously from fecal samples of the spot-billed duck (*Anas poecilorhyncha*) were used as a challenge virus. The 50% egg infective dose (EID_50_) of the H5N3 virus was calculated as described elsewhere [27]. Oropharyngeal swabs were collected form the immunized and control group chickens before and after the H5N3 virus challenge. An M2-gene specific real time PCR method was used to differentiate infected chickens from vaccinated 1 at 0 and 4 dpi, as described elsewhere [28]. At 12 weeks of age following booster immunization, chickens were challenged intra-nasally with 1 × 10^7^ EID_50_ of H5N3 virus. Chicken infection with H5N3 were monitored via oropharyngeal and cloacal swabs for 14 days after the challenge. Swabs positive for H5N3 virus were determined by qRT-PCR for the viral HA gene. After the H5N3 challenge, chickens from all groups were challenged orally with 5 × 10^6^ CFU/0.1 mL of virulent JOL394 *S*. Gallinarum. The mortality was observed daily for 2 weeks post-challenge.

### Statistical analysis

ELISA, lymphocyte proliferation assay, and viral shedding data are expressed as mean ± standard error of the mean (SEM). HAI titers are expressed as GMT ± SD. Statistical analyses were performed with SPSS 16.0 software (SPSS Inc., USA). All analyses were one-tailed, and statistical significance was identified at *p* values less than 0.05 or less than 0.005. A one-way ANOVA with post hoc Tuckey adjustments was used to analyze statistical differences in ELISA, CMI immune responses between the immunized and control groups.

## Results

### Construction and expression of HA1 protein from JOL1814

The codon-optimized synthetic HA1 gene from the H5 subtype of the influenza A virus was cloned into pMMP65, an Asd^+^ constitutive expression vector (Figure 1A). The pMMP65-HA1 gene construct was electroporated into an auxotrophic mutant of *S.* Gallinarum, JOL967 (Δ*cpxR*, Δ*lon*, and Δ*asd*). Colonies harboring the pMMP65-HA1 plasmid were confirmed by restriction enzyme analysis of isolated plasmids, and the strain was designated JOL1814. JOL1814 constitutively expressed a 36-kDa band corresponding to the HA1 protein of the H5 AIV subtype in precipitated cultures and periplasmic fractions, as shown by Western blot analysis (Figure 1B).

### In vitro delivery of HA1 antigen through JOL1814 to peritoneal macrophages

**Figure 2:**
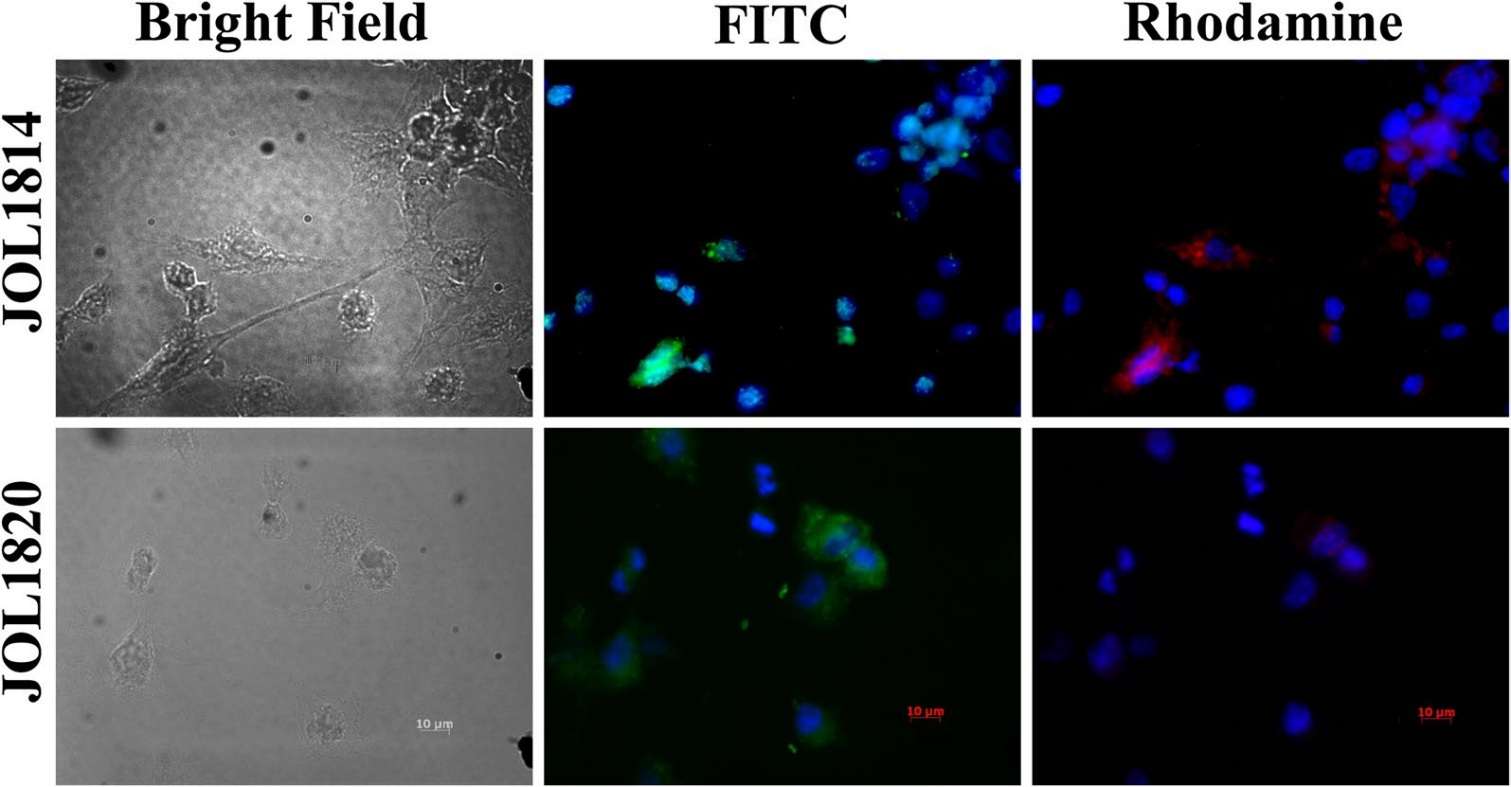
**In vitro delivery of HA1 antigen to peritoneal macrophages via JOL1814.** Harvested and cultured peritoneal macrophages were infected with JOL1814 and JOL1820 at an MOI of 100. The cells were fixed at 24 hpi and analysed for immunofluorescence by fluorescence microscopy (total magnification, 10 × 100). The JOL1814 infected peritoneal macrophages showed green and red fluorescence surrounding the blue fluorescence nuclei. The vector control JOL1820 showed only green fluorescence surrounding the blue fluorescence nuclei. DAPI: blue coloured nuclear stain; Rhodamine: tagged antibodies for localization of the HA1 antigen and FITC: tagged antibodies for localization of *S.* Gallinarum.

The in vitro macrophage invasion assay was performed to assess the delivery of the expressed HA1 proteins by JOL1814. We infected harvested chicken peritoneal macrophages with JOL1814 and a vector control strain, JOL1820. Direct plating of the lysate from infected PBMCs on BGA plates showed colonies and the JOL1814 and JOL1820 were recovered. The JOL1814 and JOL1820 colonies were confirmed by HA1 gene specific PCR. The fluorescence microscopy observation showed green fluorescence surrounding blue stained nuclei indicating uptake of JOL1814 and JOL1820 by peritoneal macrophages. Only the JOL1814-infected showed red fluorescence surrounding blue stained nuclei, suggesting the delivery of the HA1 antigen to the cytosol of the macrophage, in vitro (Figure 2).

### Specific humoral and mucosal immune responses against *S.* Gallinarum and H5 HA1 protein

**Figure 3:**
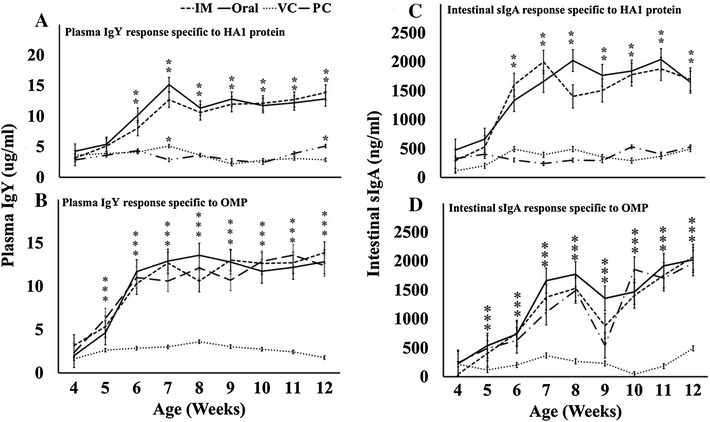
**Humoral and mucosal immune responses to LPAI H5 HA1-specific antigens and** ***S.*** **Gallinarum-specific antigens. A** The systemic IgY immune response to LPAI H5 HA1 purified protein antigen in plasma. **B** The mucosal sIgA immune response to LPAI H5 HA1 purified protein antigen in intestinal lavage. **C** The systemic IgY immune response to *S.* Gallinarum OMP protein in plasma. **D** The mucosal sIgA immune response to *S.* Gallinarum OMP protein in intestinal lavage. Oral: JOL1814 orally immunized group; IM: JOL1814 intramuscularly immunized group; VC: JOL1820 intramuscularly immunized group and PC: PBS inoculated group. *p < 0.05 when compared with PBS negative control group.

Plasma and intestinal wash samples from JOL1814-immunized and control chickens were collected and analyzed by indirect ELISA using purified HA1 and OMP proteins from H5N3 virus and *S*. Gallinarum, respectively. The plasma IgY (µg/mL) and intestinal sIgA (ng/mL) immune responses to the *Salmonella* and HA1 antigens were quantified with a standard curve plotted against a reference antibody concentration (Figure 3). The HA1-specific IgY and sIgA concentrations in chickens from all JOL1814-immunized groups were significantly higher than in the vector and PBS control groups after primary and booster immunizations (*p* < 0.05) (Figures 3A and B). The additional booster immunizations primarily maintained the plasma IgY and intestinal sIgA concentrations at a significantly high level until 12 weeks of age relative to the PBS and vector control groups (Figures 3A and B).

A bivalent immune response to the HA1 and SG antigens was present only in the JOL1814 group. Along with the HA1-specific humoral and mucosal immune response, *S.* Gallinarum-specific IgY and sIgA concentrations were also significantly elevated in the JOL1814 group after primary and booster immunizations (*p* < 0.05) (Figures 3C and D). The OMP-specific plasma IgY and intestinal sIgA immune responses again peaked compared to the primary immunization at 11 and 12 weeks of age after the booster immunization, indicating a booster effect (Figures 3C and D). The vector control group had a significant (*p* < 0.05) humoral immune response to *S.* Gallinarum that was equivalent to that of JOL181, but failed to show any response to HA1. In summary, only the bivalent vaccines elicited high plasma IgY and intestinal sIgA titers specific to H5N3 and *S.* Gallinarum, whereas the vector control elicited a specific immune response to only *S.* Gallinarum, indicating that JOL1814 is a specific bivalent vaccine.

### Cell-mediated immune (CMI) response

**Figure 4:**
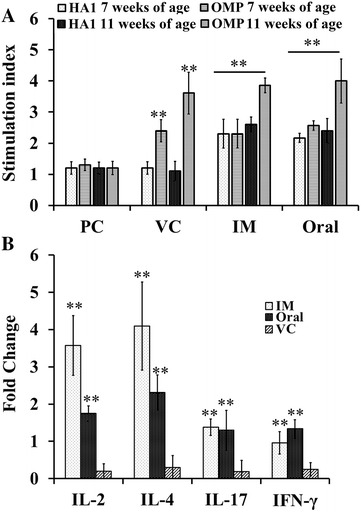
**Cytokine- and cell-mediated immune responses to LPAI H5N3 virus and soluble** ***S.*** **Gallinarum antigen. A** The stimulation index (SI) in chicken PBMC samples stimulated with (1) H5N3 HA1 purified protein antigen and (2) soluble antigen from *S.* Gallinarum. The SI was calculated by dividing the mean OD value of antigen-stimulated wells by the mean OD of media-stimulated wells. **B** Cytokine levels in stimulated PBMCs from JOL1814 immunized and PBS control chickens. PBMCs from the 1814 oral, 1814 IM, vector and PBS control groups were collected at 11 weeks of age following booster immunization and stimulated with H5N3 HA1 purified protein antigen. Cytokine levels were assessed by qPCR with gene-specific primers, and the change was calculated using 2^−∆∆Ct^. The fold change was calculated by taking the ct value for PBS control group as baseline. Each column represents the mean ± SD of six individual values. Oral: JOL1814 orally immunized group; IM: JOL1814 intramuscularly immunized group; VC: JOL1820 intramuscularly immunized group and PC: PBS inoculated group *p < 0.05; **p < 0.001 when compared with the PBS control group.

The LPA assay was performed at 7 and 11 weeks of age to assess the CMI response in immunized and control chickens. HA1 and SG antigens specifically induced simultaneous and significant induction of PBMC proliferation only in the JOL1814 group (Figure 4A). The lymphocyte proliferation responses to the HA1 antigen in the JOL1814 group revealed 2.2- and 2.4-fold increases in the stimulation indices (SI) at 7 and 11 weeks of age, respectively, over the vector and PBS controls (*p* < 0.05) (Figure 4A). In addition to the HA1-specific CMI response, JOL1814-immunized chickens also showed a specific CMI response to *S*. Gallinarum antigens (*p* < 0.05) (Figure 4A). The vector control group showed a CMI response only to *S.* Gallinarum (Figure 4A). The booster only affected the *S.* Gallinarum-specific immune response, as the SI value increased significantly by up to fourfold over the primary immunization (twofold) (*p* < 0.05). In addition, the CMI response to *S*. Gallinarum was significantly higher (*p* < 0.05) than the response to HA1 after booster immunization.

### Cytokine gene expression following stimulation of PBMCs with HA1 protein

The expression of IL-2, IL-4, IL-17 and IFN-γ in PBMCs was determined by quantitative real-time PCR following recall antigenic stimulation with the HA1 antigen. Stimulated PBMCs from the JOL1814 group exhibited significantly higher expression levels of the Th1 cytokine IFN-γ (*p* < 0.05), IL-2 (*p* = 0.05), Th2 cytokine IL-4 (*p* < 0.05), and IL-17 cytokine genes than did those from the vector and PBS control groups (Figure 4B). Within immunized group, the JOL1814 IM group showed significantly higher (*p* < 0.05) production of IL-2 and IL-4 cytokine than oral group, indicating effective stimulation of Th1 as well as Th2 arm of immunity (Figure 4B).

### Induction of functional antibody response in chickens following immunization with JOL1814

**Figure 5:**
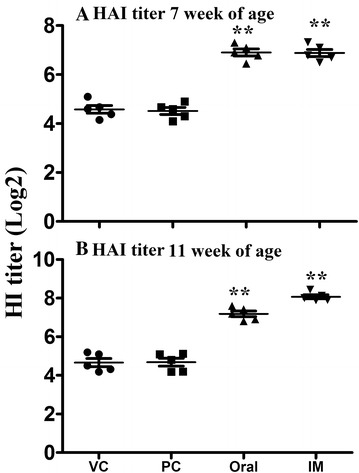
**The HAI titer in JOL1814-immunized and control groups.** HAI titers are expressed as log_2_ values of the highest dilution of serum that completely inhibited chicken RBC haemagglutination. **A** HAI titer at 7 weeks of age following post primary immunization; **B** HAI titer 11 weeks of age following post booster immunization. The SI values are presented as the mean ± SEM of each group. Significant differences in HAI titer value were estimated by calculating the GMT ± SD for each group. Oral: JOL1814 orally immunized group; IM: JOL1814 intramuscularly immunized group; VC: JOL1820 intramuscularly immunized group, and PC: PBS inoculated group. *p < 0.05 when compared with the PBS control group.

Sera collected from chickens after primary and booster immunizations were assayed for the presence of H5N3-specific functional antibodies by HI assays. As shown in Figure 5, at 7 and 11 weeks of age, chickens that were immunized orally and IM with JOL1814 developed significantly higher (*p* < 0.01) HAI antibody titers against the LPAI H5N3 influenza virus. The HAI titers expressed as a geometric mean of the virus neutralization antibody titers (GMTs) in the JOL1814-immunized oral and IM groups were 6.45 ± 0.28 and 6.78 ± 0.29, respectively, at 7 weeks of age (*p* < 0.01) (Figure 5A). After booster immunization, the HAI titer in the JOL1814 oral and IM groups increased significantly over primary immunization (*p* < 0.05) (Figure 5B). The increases in the GMT for HAI following booster immunization in the oral and IM groups were 7.43 ± 0.30 and 8.45 ± 0.29, respectively (*p* < 0.01) (Figure 5B). The HAI titers in the PBS and vector control groups did not change significantly: 4.2 ± 0.28 and 4.15 ± 0.23 at 7 and 11 weeks of age, respectively (Figure 5).

### Protective efficacy against H5N3 LPAI virus and DIVA

**Figure 6:**
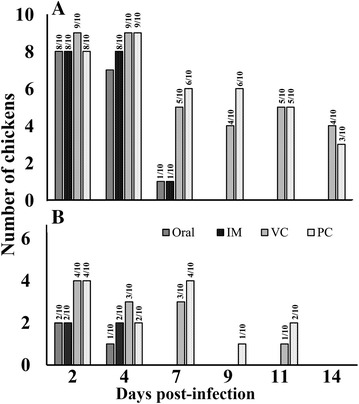
**Protective efficacy against LPAI H5N3 virus challenge in JOL1814-immunized and control group chickens.** Ten chickens from each group were infected with LPAI H5N3 virus. Oropharyngeal and cloacal swabs were collected at the indicated time points after infection (dpi). The swabs were evaluated for presence of H5N3 challenge virus by qRT-PCR. **A** Number of birds positive for LPAI H5N3 infection in oropharyngeal swabs. **B** Number of birds positive for LPAI H5N3 infection in cloacal swabs. The number above each column indicates the number of positive samples per total number of analyzed swab samples.

The chickens were challenged intranasally with LPAI virus to evaluate protection conferred to chickens by JOL1814 immunization. Despite intranasal challenge with 10^7^ EID_50_ of H5N3 virus, no adverse clinical signs were observed in the JOL1814 or control chickens. In the absence of any adverse clinical signs, at 2 days post-infection (dpi) more than 80% of chickens from the immunized and control groups shown presence of H5N3 duck isolate virus in the oropharyngeal swab indicating infection of chickens. All the oropharyngeal swab samples collected before challenge at 0 dpi from JOL1814 immunized as well as control group chickens were found to be negative for amplification of M2 gene, whereas, post-challenge at 4 dpi 70, 80 and 90% swabs from JOL814 oral, JOL1814 IM, JOL1820 vector control and PBS control were tested positive for M2 gene amplification (Table 2). The successful infection of chickens with H5N3 LPAI virus was supported by the observation that H5N3 virus was isolated from oropharyngeal and cloacal swabs at 2 dpi. The JOL1814 oral and IM groups completely cleared the H5N3 LPAI virus at 7 and 4 dpi, respectively, as determined by oropharyngeal and cloacal swabs (Figure 6). Contrary to the JOL1814 group, control chickens remained infected and shed H5N3 LPAI virus in oropharyngeal and cloacal swabs until 11 and 14 dpi, respectively (Figure 6).

**Table 2:** **DIVA potential of the** ***S.*** **Gallinarum LBVs**

Groups	No. of chickens positive for H5N3 virus	
	Before challenge (0 dpi)	Post challenge (4 dpi)
JOL1914 Oral	0	7
JOL1814 IM	0	8
JOL1820 VC	0	9
PBS control	0	9

The M2e gene based qRT-PCR test were used to detect presence of H5N3 virus in the Oropharyngeal swabs collected from inoculated and challenged chickens at 0 dpi (before challenge) and 2 dpi, respectively.

### Evaluation of protective efficacy against lethal *S.* Gallinarum challenge in immunized and control chickens

**Figure 7:**
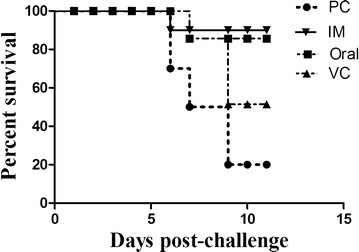
**Mortality rates of JOL1814-immunized and control group chickens post** ***S.*** **Gallinarum challenge.** The x-axis depicts the days for which the mortality rate was observed in chickens after challenge with virulent *S.* Gallinarum JOL394. The y-axis represents the percent survival rate. Until day 6 after challenge, no death was reported. Oral: JOL1814 orally immunized group; IM: JOL1814 intramuscularly immunized group; VC: JOL1820 intramuscularly immunized group; and PC: PBS inoculated group.

The survival rate following lethal challenge with wild-type (WT) *S*. Gallinarum, JOL394, was evaluated to assess protection in JOL1814-immunized, vector, and control chickens until 14 days after the challenge. After the challenge, a few chickens from the JOL1814 and vector groups were depressed until 5 dpi, but recovered thereafter. The PBS control chickens had decreased feed intake, severe depression and subsequent mortality. The JOL1814 immunized and vector control groups showed significantly higher protection against the *S.* Gallinarum challenge. The oral and IM immunized groups had 10% mortality compared to 30 and 80% mortality in the vector and PBS control groups, respectively (Figure 7). All chickens were euthanized at 14 days after the challenge, and pathognomonic legions specific to *S*. Gallinarum infection were assessed on the liver and spleen. The livers and spleens from the PBS control chickens were hemorrhagic and enlarged, whereas JOL1814 chickens had normal livers and spleens.

## Discussion

A strain matched and cost-effective vaccine against different H5 subtypes of LPAI viruses with DIVA capacity is needed to control LPAI virus outbreaks and thus prevent emergence of HPAI viruses [3, 7]. Recently, attenuated *Salmonella*-based LBV systems that can be engineered to deliver a DNA vaccine for AIV have shown promising results [12]. A potential limitation of DNA vaccine delivery through *Salmonella* is the inability to stably maintain the plasmid inside the delivery vehicle. To overcome this limitation of a DNA vaccine, we constructed JOL1814, an attenuated auxotrophic mutant of *S*. Gallinarum expressing the HA1 antigen of the AIV H5 subtype virus on an Asd^+^ plasmid, and used it to immunize chickens. The auxotrophic *S.* Gallinarum mutant, a balanced-lethal host-vector system was previously constructed by deletion of the aspartate-semialdehyde dehydrogenase (asd) from wild-type strain and which can be functionally complemented with Asd^+^ plasmid [29]. The ability to infect and multiply inside macrophages makes *S.* Gallinarum an effective LBV for expressing and delivering heterologous antigens into APC [30, 31]. In a previous study have shown the invasion potential of attenuated *S.* Gallinarum (*∆lon* and *∆cpxR*) in peritoneal macrophages and quantified the results as colony forming unit per mL (CFU/mL), the attenuated *S*. Gallinarum (3.81 ± 0.03 CFU/mL) showed significantly higher viable cell counts than wild type (3.32 ± 0.03 CFU/mL), indicating more invasion potential of *S*. Gallinarum vaccine strain [32]. Further, the results of fluorescent microscopy showed that JOL1814 effectively delivered the HA1 antigen to the cytoplasm of infected peritoneal macrophages in vitro, suggesting that *S.* Gallinarum based JOL1814 LBVs was able to deliver the expressed HA1 antigenic protein to the APCs for further MHC presentation (Figure 2). Previously, we demonstrated that an attenuated *Salmonella typhimurium* vector-based influenza vaccine had efficacy for pandemic preparedness against H1N1 influenza viruses. Here, we further extend this approach by engineering attenuated *S.* Gallinarum to provide bivalent protection against fowl typhoid and LPAI H5N3 viruses.

Chickens immunized with JOL1814 by oral and IM routes had significantly induced levels of plasma IgY and intestinal sIgA antibodies specific to H5 and *S.* Gallinarum antigens. JOL1814 immunized chickens had highest HA1 specific (*p* < 0.05) plasma IgY and intestinal sIgA responses at 7 and 8 weeks of age, respectively, after primary immunization. Immunizing chickens with a JOL1814 booster dose failed to significantly increase the HA1 specific plasma IgY and intestinal sIgA concentrations compared to primary immunization. Furthermore, JOL1814 also induced a significant level of HAI titer against LPAI H5N3 virus. Live *Salmonella* vaccines can colonize the lymphoid organs and Peyer’s patches at high concentrations and stimulate both mucosal and humoral immune responses [18]. Mucosal sIgA and systemic IgY antibodies specific to H5 HA1 and *S.* Gallinarum antigens provide immediate, early, and neutralizing immunity against invading *S.* Gallinarum and influenza viruses, respectively [33, 34]. Systemic plasma IgY antibodies clear *Salmonella* infections from the blood by opsonizing the *Salmonella* and promoting its phagocytosis by antigen-presenting cells [35]. Plasma IgY and neutralizing antibody-based immunity are sufficient to prevent systemic infection, but were inadequate to protect against upper respiratory tract infection in mice and ferrets [36, 37]. To protect chickens against both *S.* Gallinarum and H5N3 influenza virus infection, sIgA antibodies act as an immunological barrier at mucosal level, thus limiting the spread to internal organs [38, 39]. Collectively, these results indicate that JOL1814 can induce humoral and mucosal immunity via oral and IM immunization. In addition to humoral and mucosal immunity, cell-mediated immunity is required for chickens to completely recover from *S.* Gallinarum and influenza infections. The cellular immune responses induced by influenza viruses are necessary for viral clearance from the lungs and, therefore, are critical for chickens to recover from influenza virus infections [40]. Although the HA1-specific SI value at 11 weeks of age for the JOL1814 group did not increase significantly over 7 weeks of age (*p* < 0.05) (Figure 4A), a booster immunization maintained the HA1-specific CMI response at a level equivalent to primary immunization. In present study, it was observed the booster immunization was able to maintain and significantly elevate the cell mediated immunity against influenza and *S.* Gallinarum, respectively. Further, enhanced cellular immunity contributes significantly to both protecting chickens from primary *Salmonella* infection and clearing *S.* Gallinarum infection from macrophages [41]. Our results show that JOL1814 immunization significantly increased cellular immunity to *S.* Gallinarum and LPAI H5N3 virus.

Oral and IM JOL1814-immunized chickens had significantly higher levels of IL-2, IL-4, IL-17, and IFN-γ mRNA in stimulated PBMCs. The IFN-γ and IL-2 cytokines which is produced by stimulated natural killer cells, macrophages, CD4+ and CD8+ T cells, controls the generation of antigen-specific CMI, whereas IL-4 cytokines are generally produced by Th2 cells and mediate the generation of an antigen-specific antibody response [42]. Recent findings have shown that IL-17 cytokines are important for inducing innate and adaptive host responses and contribute to maintaining barrier function against pathogens in the host mucosa [43]. The PBMCs isolated from the immunized and control group chickens were stimulated only with purified HA1 antigen, hence the significant increase in fold change in IL-2, IL-4, IL17 and IFN-γ were observed only in JOL1814 immunized group, whereas, the JOL1820 vector control showed cytokine response equivalent to PBS control, indicating generation of HA1 antigen specific cytokine response. In previous reports, the PBMC pulsed with soluble antigen fraction of *S.* Gallinarum resulted in significant increase in IL-6 cytokine in the immunized chickens [24]. Overall, the cytokine analysis of the stimulated PBMC culture correlates with augmented immunogenicity specific to JOL1814 immunization in chickens.

The protective efficacy of the JOL1814 bivalent vaccine was evaluated by viral challenge with *S*. Gallinarum and LPAI H5N3. The LPAI H5N3 virus infected chickens and was continuously shed into the environment through the nasal and cloacal routes [44]. In this study, JOL1814-immunized chickens were protected against LPAI H5N3 virus infection. Both oral and IM JOL1814-immunized chickens cleared H5N3 virus infection by day 7 pi. Compared to the immunized group at 14 dpi, 30 and 40% challenged chickens from PBS control and vector control group were positive for presence of LPAI H5N3 virus in oropharyngeal swabs (Figure 6A). In a previous study, the efficacy of an LBV vaccine against LPAI viruses was evaluated in a mouse model [37]. Oral inoculation of BALB/c mice with *Lactobacillus plantarum* expressing the HA gene of LPAI H9N2 completely protected against lethal challenge with mouse-adapted H9N2 virus [45]. Further, after challenging H5N3-infected chickens with a virulent dose of *S.* Gallinarum (WT), PBS control chickens had 80% mortality with profound liver and spleen lesions. On the other hand, the JOL1814 group and JOL1820 (VC) chickens were well protected against virulent *S*. Gallinarum challenge, with only 10–30% mortality. Taken together, our results suggest that chickens in the JOL1814 group were significantly protected from LPAI H5N3 infection and shedding and survived virulent challenge with *S.* Gallinarum.

In conclusion, for the first time, we showed that an attenuated *S*. Gallinarum-based LBV for influenza virus is an efficient bivalent vaccine candidate against fowl typhoid and LPAI H5N3 virus in chickens. Immunizing chickens with JOL1814 significantly induced humoral, mucosal, and cell-mediated immunities against H5N3 HA1 and *S.* Gallinarum antigens, and this induced immunogenicity significantly reduced H5N3 viral infection and *S.* Gallinarum-induced mortality in chickens. Future studies are needed to determine the protective effect of JOL1814 against heterologous LPAI H5 subtype virus challenge.

## Abbreviations

AIV: avian influenza virus
LPAI: low pathogenic avian influenza
HPAI: high Pathogenicity avian influenza
FT: fowl typhoid
LBVs: live bacterial vaccine vectors
DIVA: differentiate infected from vaccinated animals
LB: Luria–Bertani
ELISA: indirect enzyme-linked immunosorbent assay
PBMC: peripheral blood mononuclear cells
HI: haemagglutination inhibition
CMI: cell mediated immunity
